# Transcriptomic responses and molecular mechanisms of glucagon-regulated glucose metabolism in Japanese flounder (*Paralichthys olivaceus*)

**DOI:** 10.3389/fphys.2026.1825972

**Published:** 2026-04-16

**Authors:** Ting Zhang, Hongquan Wang, Tiaoyi Xiao, Mengxi Yang, Wenbing Zhang

**Affiliations:** 1 Hunan Engineering Technology Research Center of Featured Aquatic Resources Utilization, Fisheries College, Hunan Agricultural University, Changsha, China; 2 The Key Laboratory of Aquaculture Nutrition and Feeds (Ministry of Agriculture and Rural Affairs), The Key Laboratory of Mariculture (Ministry of Education), Ocean University of China, Qingdao, China

**Keywords:** glucagon, glucose metabolism, insulin, *Paralichthys olivaceus*, TRIB3

## Abstract

Glucagon is a key hormone regulating gluconeogenesis and glucose homeostasis in mammals, yet its regulatory mechanisms in glucose metabolism in carnivorous fish remain incompletely understood. To systematically investigate glucagon-mediated glucose metabolism in Japanese flounder (*Paralichthys olivaceus*), liver samples were collected before glucagon injection (0 h) and at 1 h and 6 h post-injection for transcriptome sequencing (RNA-seq). Transcriptome analysis identified numerous differentially expressed genes involved in glucose and energy metabolism. In total, 507, 1458, and 709 differentially expressed genes were detected in the comparisons of 0 h vs 1 h, 1 h vs 6 h, and 0 h vs 6 h, respectively. KEGG enrichment analysis showed that glucagon activated pathways related to glucagon signaling, insulin resistance, FoxO signaling, and energy metabolism, including AMPK and PPAR pathways, suggesting that glucagon rapidly stimulates gluconeogenesis. At 6 h post-injection, genes involved in glycolysis and glucose transport were upregulated, whereas key gluconeogenic genes were downregulated, indicating attenuation of glucagon-induced metabolic responses. Further analysis showed that glucagon suppressed the insulin-mediated PI3K/AKT signaling pathway. Among the candidate genes, SOCS3 and TRIB3 were upregulated and may serve as key regulators linking glucagon and insulin signaling. Functional experiments further showed that knockdown of TRIB3 reduced glucose levels in hepatocyte culture medium and increased the expression of insulin signaling-related genes. Overall, glucagon regulates glucose metabolism in Japanese flounder by promoting gluconeogenesis while suppressing insulin signaling, providing transcriptomic insights into endocrine regulation in carnivorous fish.

## 1 Introduction

Glucagon plays a pivotal role in maintaining glucose homeostasis in vertebrates (Moon, 1998; Lin and Accili, 2011). In mammals, glucagon stimulates hepatic gluconeogenesis mainly through activation of the cAMP/PKA and PLC/Ca^2+^ signaling pathways. This process promotes the expression of key gluconeogenic enzymes, such as phosphoenolpyruvate carboxykinase (PCK1) and glucose-6-phosphatase (G6PC) (Hanson and Reshef, 1997; Yoon et al., 2001; Jiang and Zhang, 2003; Wang et al., 2012). Dysregulation of glucagon secretion is closely associated with metabolic disorders. Elevated circulating glucagon levels are frequently reported in individuals with type 2 diabetes and are thought to contribute to the maintenance of hyperglycemia (Unger, 1985; Burcelin et al., 1996). Compared with mammals, teleost fish generally display a limited capacity to utilize dietary carbohydrates, particularly in carnivorous species (Moon, 2001). After feeding high-carbohydrate diets or glucose administration, many fish species exhibit prolonged hyperglycemia, indicating impaired glucose tolerance (Polakof et al., 2012; Lin et al., 2018). Previous research indicates that excessive hepatic glucose production plays a crucial role in fish glucose homeostasis. For instance, comparative studies between the herbivorous species grass carp (*Ctenopharyngodon idella*) and the carnivorous long-snouted catfish (*Leiocassis longirostris*) revealed that carnivorous fish exhibit a weaker ability to suppress hepatic gluconeogenesis following carbohydrate feeding (Su et al., 2020). Similarly, in European seabass (*Dicentrarchus labrax* L.), endogenous glucose production contributes substantially to circulating glucose levels (Viegas et al., 2011). These physiological characteristics suggest that increased hepatic glucose production may represent a major factor underlying the relatively poor carbohydrate utilization observed in carnivorous fish, in which glucagon acts as a key regulatory hormone (Lin et al., 2018; Yan et al., 2020).

The metabolic effects of glucagon extend beyond stimulation of gluconeogenesis (Perry et al., 2020). In mammalian liver, glucagon promotes glycogenolysis and gluconeogenesis while suppressing glycolysis, thereby coordinating multiple pathways involved in hepatic glucose production (Biazi et al., 2018). Similar metabolic responses have also been reported in fish species, where glucagon stimulates glycogen breakdown and inhibits glycolytic activity in liver (Forbes et al., 2019; Yan et al., 2020). In addition, glucagon signaling can interact with insulin pathways to regulate glucose metabolism. For example, glucagon has been shown to influence the PI3K/AKT signaling cascade and thereby modulate insulin-mediated metabolic responses (Rodgers, 2012). In high-fat-diet–induced mouse models, elevated glucagon levels enhance hepatic glucose production through activation of FoxO1 and inhibition of the AKT pathway (Liu et al., 2017). Furthermore, glucagon contributes to metabolic adaptation by promoting skeletal muscle protein degradation, which supplies amino acid for gluconeogenesis. It also stimulates hepatic fatty acid oxidation to provide energy for glucose production (Adeva-Andany et al., 2019). These findings indicate that glucagon participates in the integrated regulation of hepatic glucose metabolism rather than acting solely as a gluconeogenic hormone (Yan et al., 2020; Janah et al., 2019).

Regulators of insulin signaling may represent important mediators linking glucagon signaling to hepatic glucose metabolism. Among them, Tribbles pseudokinase 3 (TRIB3) is a key regulator that interacts with AKT and suppresses its phosphorylation, which consequently attenuates PI3K/AKT signaling (Du et al., 2003). In mammals, increased TRIB3 expression has been associated with insulin resistance and enhanced hepatic gluconeogenesis through activation of FoxO1-dependent transcription (Marinho et al., 2015; Oberkofler et al., 2010). Moreover, under metabolic stress conditions, TRIB3 promotes hepatic glucose production by suppressing AKT signaling and altering downstream metabolic pathways (Marinho et al., 2015). Although the role of TRIB3 in mammalian glucose metabolism has been extensively investigated, its potential involvement in glucagon-mediated metabolic regulation in fish remains largely unknown.

Japanese flounder (*Paralichthys olivaceus*) is a commercially important carnivorous marine fish. Deng et al. (2018) reported that diets containing high levels of carbohydrates significantly impaired growth performance and glucose tolerance in this species. These physiological characteristics make Japanese flounder a suitable model for investigating the endocrine regulation of glucose metabolism in carnivorous fish. Our previous work demonstrated that glucagon promotes hepatic gluconeogenesis in Japanese flounder through activation of the GCGR/PKA/CREB/PGC-1α signaling pathway (Yang et al., 2023). However, whether glucagon regulates glucose metabolism through additional molecular mechanisms in this species remains unclear. Therefore, in the present study, synthetic Japanese flounder glucagon was administered, and liver transcriptome sequencing (RNA-seq) was conducted to systematically identify glucagon-responsive genes involved in glucose metabolism. In addition, knockdown experiments were conducted in the primary hepatocytes to explore the potential role of candidate regulators in glucagon-mediated metabolic regulation. This study provides novel insights into the endocrine regulatory network governing glucose metabolism in carnivorous fish and helps clarify the molecular basis of glucose intolerance, which may ultimately facilitate strategies for improving carbohydrate utilization in aquaculture species.

## 2 Materials and methods

### 2.1 Experimental animals

Japanese flounder with an average body weight of 36.03 ± 5.21 g were obtained from Qingyuan Marine Biotechnology Co., Ltd. (Qingdao, Shandong, China). Fish were randomly distributed into aerated indoor circulating water tanks (200 L). Before the experiment, the fish were acclimated for two weeks and fed a commercial diet (Saigelin Bioengineering Co., Ltd., Qingdao, Shandong, China).

### 2.2 Intraperitoneal injection of glucagon

Japanese flounder glucagon was synthesized as previously described (Yang et al., 2023). Before injection, fish were fasted for 24 h. Three tanks were assigned to each treatment group (each sampling time point). After anesthesia with eugenol, fish in the glucagon group were intraperitoneally injected with glucagon at a dose of 150 ng g^−1^ body weight in a volume of 100 μL. Fish in the control group received an equal volume of 0.9% saline solution. Based on previously obtained plasma glucose results (Yang et al., 2024), where glucose levels peaked at 1 h after glucagon injection and significantly decreased to baseline by 6 h, liver samples were collected before injection (0 h) and at 1 h and 6 h post-injection for transcriptome analysis. At each sampling point, three fish were randomly collected from each tank and pooled to form one composite sample per tank for RNA extraction. Therefore, each treatment group included three biological replicates. In total, nine RNA-seq samples were obtained. Fish were anesthetized and euthanized by cranial percussion. Blood samples were collected, and liver tissues were excised and stored at −80 °C for subsequent analyses.

### 2.3 RNA-seq sample preparation

Total RNA was extracted and quality-assessed by Majorbio Bio-Pharm Technology Co., Ltd. (Shanghai, China). Qualified RNA samples were fragmented and reverse-transcribed into cDNA for library construction. After quality validation, the libraries were sequenced using high-throughput sequencing technology.

### 2.4 Transcriptome analysis

RNA sequencing and data processing were performed by Majorbio Bio-Pharm Technology Co., Ltd. Raw reads were filtered to obtain clean reads for downstream analysis. Differential gene expression analysis was conducted using DESeq2, with thresholds of |log_2_ fold change| ≥ 1 and adjusted *P* value (padj) < 0.05. Gene Ontology (GO) and Kyoto Encyclopedia of Genes and Genomes (KEGG) databases were used for functional annotation and enrichment analysis. The reference genome used for alignment was *Paralichthys olivaceus* (Flounder_ref_guided_V1.0; genome ID: 10840).

### 2.5 Isolation and culture of primary hepatocytes

Primary hepatocytes were isolated from Japanese flounder liver and cultured as previously described (Yang et al., 2023). Briefly, liver tissues were aseptically excised, washed with cold PBS containing penicillin-streptomycin, and minced into small fragments. The tissues were digested with 0.25% trypsin–EDTA at 23 °C. The cell suspension was filtered and centrifuged at 1000 g for 5 min. After treatment with red blood cell lysis buffer and washing with PBS, the hepatocytes were resuspended in complete DMEM/F12 medium and seeded into six-well plates. Cells were maintained at 23 °C. Hepatocytes reaching 80-90% confluence were used for subsequent experiments.

### 2.6 TRIB3 gene knockdown

Three small interfering RNAs (siRNA-773, siRNA-1357, and siRNA-1509) targeting TRIB3 and a nonspecific negative control siRNA (NC-siTRIB3) were designed and synthesized by Sangon Biotech Co., Ltd. (Shanghai, China). The three siTRIB3 constructs were used as experimental treatments, while NC-siTRIB3 served as the negative control. Untreated cells were used as the control group. The sequences are listed in **Table 1**. For transfection, 5 μg siRNA and 3.75 μL Lipofectamine^®^ 3000 (Invitrogen, CA, USA) were co-transfected into primary hepatocytes in six-well plates. Cells were collected 24 h after transfection, and the knockdown efficiency was evaluated by quantitative real-time PCR (qRT-PCR). Only siRNA constructs achieving a knockdown efficiency greater than 50% were used for subsequent functional experiments.

**Table 1:** siRNA oligo sequences for TRIB3 in Japanese flounder.

Primer	Sense (5’-3’)	Antisense (5’-3’)
siRNA-773	CAGCCCAAUCUCAAAUGUGUCUGAA	UUCAGACACAUUUGAGAUUGGGCUG
siRNA-1357	UGCGUCCUCCUUCACGGUAACCAUA	UAUGGUUACCGUGAAGGAGGACGCA
siRNA-1509	CAUGCUGAUUGGACGGUACCCGUUU	AAACGGGUACCGUCCAAUCAGCAUG
NC	CAGAACUCUAACGUAUGUUCCCGAA	UUCGGGAACAUACGUUAGAGUUCUG

### 2.7 Combined treatment of glucagon and siTRIB3

Three experimental groups were established: control group (DMSO + NC), glucagon treatment group (1 μM glucagon + NC), and glucagon plus siTRIB3 group (1 μM glucagon + siTRIB3). Each group contained three replicates. In brief, primary hepatocytes were first transfected with siNC or siTRIB3. Two hours after transfection, glucagon or an equal volume of DMSO was added to the culture medium. Cells were further cultured for 22 h before harvesting for subsequent analyses.

### 2.8 Measurement of glucose in culture medium

After treatment, the culture supernatants were collected. Glucose concentration in the medium was measured using a commercial glucose assay kit based on the glucose oxidase method (Peng et al., 2022).

### 2.9 qRT-PCR

Total RNA was reverse-transcribed into cDNA using a reverse transcription kit according to the manufacturer’s instructions. The qRT-PCR was conducted using a SYBR Green-based detection system (Vazyme Biotech, China) on a Quant Studio 5 Real-Time PCR system (Applied Biosystems, USA). The primer sequences used for gene expression analysis are listed in **Table 2**. *β-actin* was used as the internal reference gene for normalization. Relative gene expression levels were calculated using the 2^^−ΔΔCt^ method. Prior to analysis, primer amplification efficiency was evaluated using serial dilutions to ensure reliable quantification.

**Table 2:** Primer sequences used for qPCR analysis.

Primer	Sense (5’-3’)	Antisense (5’-3’)
*gcg*-q-F	CGGACTCCTGCTTCTCAT	MW727222
*gcg*-q-R	TTTGCTGCCTTGTCTTGC	
*socs3*-q-F	TCCAACTTCACCCCACATCG	XM_020094150
*socs3*-q-R	TCGCAGGTTCTTGGTTCCTC	
*g6pc*-q-F	TACCCCGTGACCTGTGAGAC	KY742717
*g6pc*-q-R	TTGGTGGATTTCTTGCTTCC	
*pck1*-q-F	ACCAGGGACCAGAAGGACA	MN173838
*pck1*-q-R	TGGCGACCACGTAGGGAGA	
*ins*-q-F	CCTCTGCTGGGTTTCCTTCC	XM_020108848
*ins*-q-R	TGCTCCACGATTCCTCGCTT	
*gck*-q-F	TCCTGTCATCCCTGGGTGTT	MN173836
*gck*-q-R	CTGCGTCGCTCCCTCATT	
*irs1*-q-F	CCCACTTAGGAAAAGCAGAG	KY763982
*irs1*-q-R	AGTACAGGAACGGAAGGATC	
*glut4*-q-F	AGGACGTACTGGGTCGATCA	XM_020095562
*glut4*-q-R	GGTGACAGATGCGTCTAGGG	
*sgk1*-q-F	AACCCAGCGACTTCCACTT	XM_020091919
*sgk1*-q-R	TGACATTCTTCAGCAGCACAT	
*acaca*-q-F	ACGGCGGACACGTCTTCT	MN173844
*acaca*-q-R	GCACTCTGCTCGGGTCAT	
*mknk1*-q-F	CTGGCAGATGAGGTTCTTG	XM_020081359
*mknk1*-q-R	GCAAACTCCTGTCCGTTCT	
*calm2*-q-F	ACCATCACCACCAAAGAGC	XM_020092160
*calm2*-q-R	GTCCCATTTCCATCAGCAT	
*trib3*-q-F	TTACGTCAGCCCTGAGTTGC	XM_020096491
*trib3*-q-R	CTGAAACGGGTACCGTCCAA	
*pi3k*-q-F	GCTCATCAACCACTATCGC	KY763984
*pi3k*-q-R	TGTCTTCTTTCACCACCTG	
*akt2*-q-F	CATCCCTTTCTAACAACACTA	KY763980
*akt2*-q-R	CTGTAAACAACATTGCGTGA	
*β-actin*-F	GGAAATCGTGCGTGACATTAAG	HQ386788
*β-actin*-R	CCTCTGGACAACGGAACCTCT	

gcg, proglucagon; socs3, suppressor of cytokine signaling 3; g6pc, glucose-6-phosphatase catalytic subunit; pck1, phosphoenolpyruvate carboxykinase 1; ins, insulin; gck, glucokinase; irs1, insulin receptor substrate 1; glut4, solute carrier family 2 member 4; sgk1, serum/glucocorticoid regulated kinase 1; acaca, acetyl-CoA carboxylase alpha; mknk1, MAPK interacting serine/threonine kinase 1; calm2, calmodulin 2; trib3, tribbles pseudokinase 3; pi3k, phosphatidylinositol-4,5-bisphosphate 3-kinase; akt2, AKT serine/threonine kinase 2.

### 2.10 Statistical analysis

All data are presented as mean ± standard error (Mean ± SE). Statistical analyses were performed using GraphPad Prism 9. Differences among multiple groups were analyzed using one-way analysis of variance (ANOVA) followed by Tukey’s multiple comparison test. For comparisons between two groups, an unpaired Student’s *t*-test was used. Differences were considered statistically significant at *P* < 0.05.

## 3 Results

### 3.1 Overview of transcriptome sequencing data

**Figure 1:**
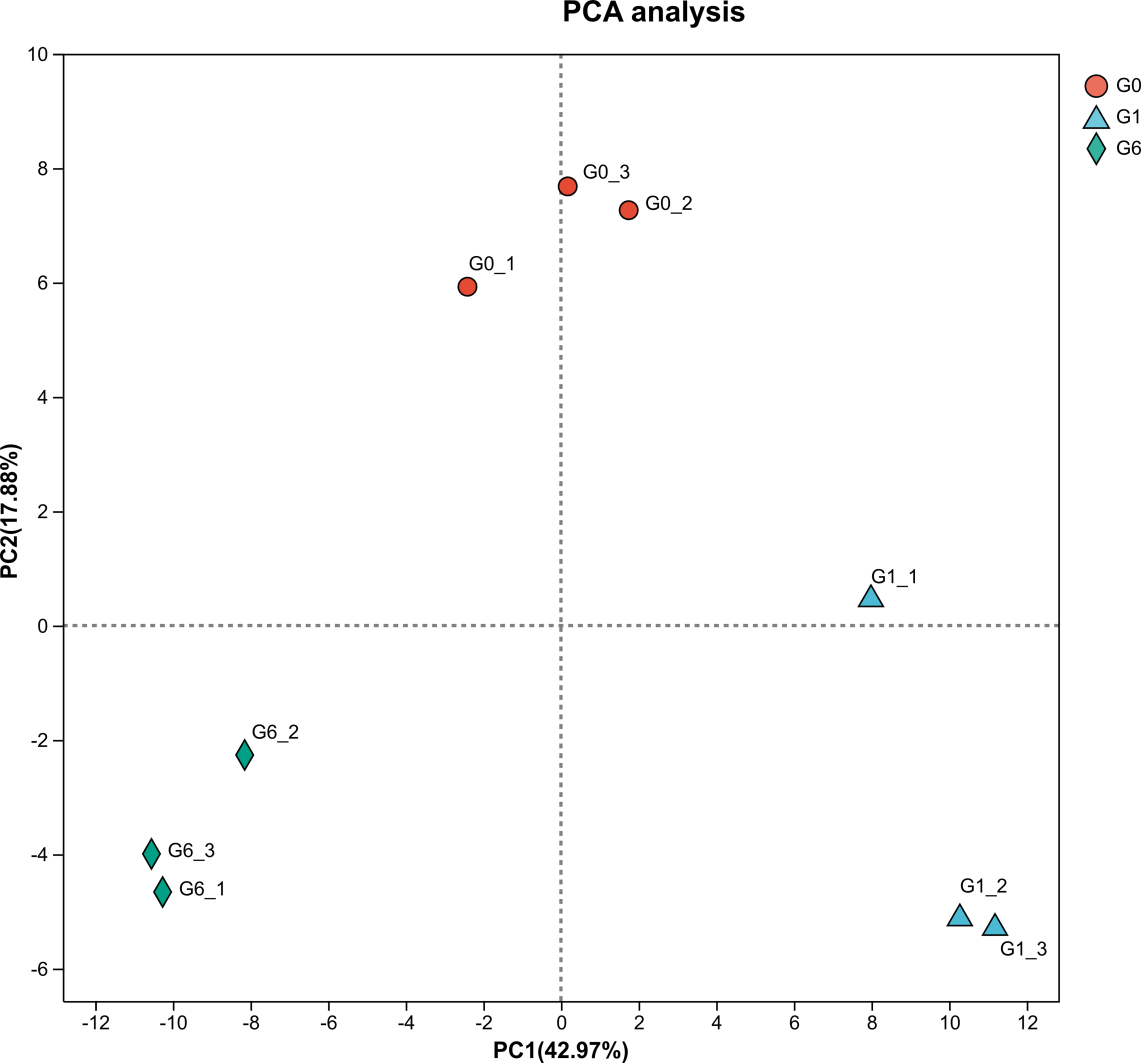
Principal component analysis. Each point represents one biological replicate. G0, G1, and G6 indicate livers collected at 0 h, 1 h, and 6 h after glucagon injection. PC1 and PC2 explain 42.97% and 17.88% of the total variance, respectively.

Principal component analysis (PCA) showed clear separation among samples collected at different time points, while the three biological replicates within each group clustered closely together (**Figure 1**). The sequencing output statistics are summarized in **Table 3**. For each sample, the amount of clean data exceeded 6.97 Gb. Clean reads were aligned to the reference genome of *Paralichthys olivaceus*, with mapping rates ranging from 89.82% to 91.06%, indicating high sequencing quality and reliability. A total of 23,213 expressed genes were detected in this study, including 21,641 known genes and 1,572 novel genes. In addition, 39,205 transcripts were identified, consisting of 30,919 known transcripts and 8,286 novel transcripts.

**Table 3:** Sequencing information for different samples of *Paralichthys olivaceus* with glucagon injection.

Sample	Raw reads	Clean reads	Clean reads ratio (%)	Mapping rate (%)
G0_1	52800240	52061894	98.6	90.3
G0_2	52387174	51548676	98.4	90.67
G0_3	52913746	52011746	98.3	90.56
G1_1	51026080	50389036	98.75	90.41
G1_2	58112908	57241628	98.5	90.56
G1_3	50789152	49758832	97.97	89.82
G6_1	52695926	52099852	98.87	91.06
G6_2	47902028	47374884	98.9	90.71
G6_3	48731328	48222022	98.95	90.72

G represents liver samples. G0, G1, and G6 indicate samples collected before glucagon injection (0 h) and at 1 h and 6 h after injection, respectively. The numbers following the underscore represent biological replicates. Clean reads were obtained after removing adaptor sequences, reads containing more than 10% ambiguous bases (N), and low-quality reads. The mapping rate indicates the percentage of clean reads successfully aligned to the reference genome.

### 3.2 Identification of differentially expressed genes

**Figure 2:**
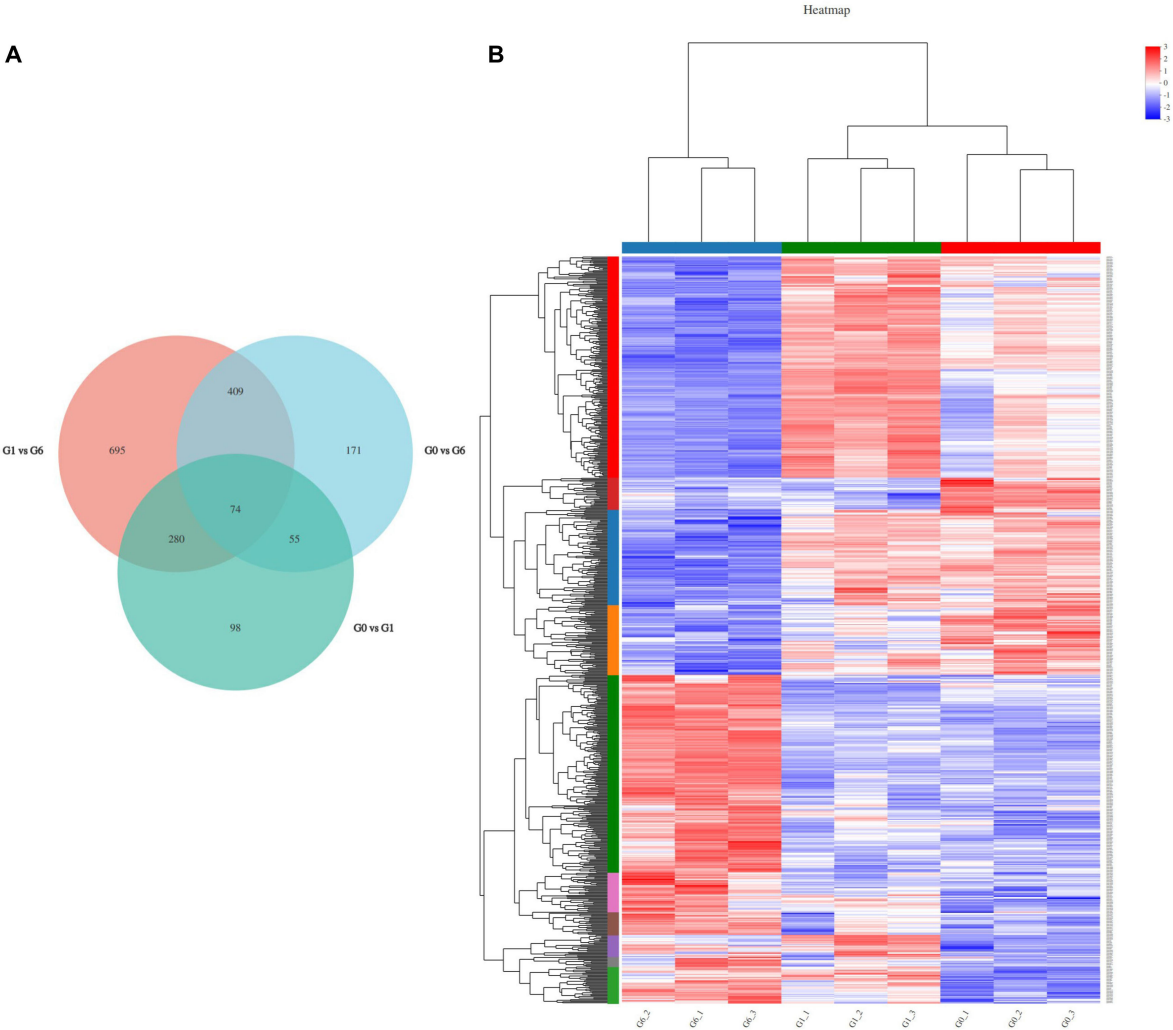
Venn diagram **(A)** and hierarchical clustering heatmap of differentially expressed genes (DEGs) **(B)**. **(A)**, Venn diagram showing the overlap of DEGs among different comparisons. **(B)**, Heatmap showing hierarchical clustering of DEGs. Each column represents a sample and each row represents a gene. Colors from blue to red indicate low to high expression levels.

The summary of differentially expressed genes (DEGs) is shown in **Table 4**. Three comparison groups were analyzed: 0 h vs 1 h (G0 vs G1), 1 h vs 6 h (G1 vs G6), and 0 h vs 6 h (G0 vs G6). Using the criteria of *P*-adjust < 0.05 and |log_2_FC| ≥ 1, a total of 1,782 DEGs were identified in the liver of Japanese flounder following glucagon injection. Compared with the 0 h group, 507 DEGs were identified at 1 h post-injection, including 388 upregulated genes and 119 downregulated genes. Compared with the 1 h group, 1,458 DEGs were identified at 6 h post-injection, including 466 upregulated genes and 992 downregulated genes. In the comparison between 6 h and 0 h, 709 DEGs were detected, including 312 upregulated genes and 397 downregulated genes. The overlap among DEGs from the three comparison groups was visualized using a Venn diagram (**Figure 2A**). Furthermore, hierarchical clustering analysis was performed to evaluate the similarity of gene expression patterns (**Figure 2B**). The results showed that samples within the same time point displayed similar expression profiles, whereas significant differences were observed among different time points.

**Table 4:** Summary of DEGs identified in different comparisons.

Comparison	Total DEGs	Upregulated	Downregulated
G0 vs G1	507	388	119
G1 vs G6	1458	466	992
G0 vs G6	709	312	397

### 3.3 GO functional enrichment analysis

**Figure 3:**
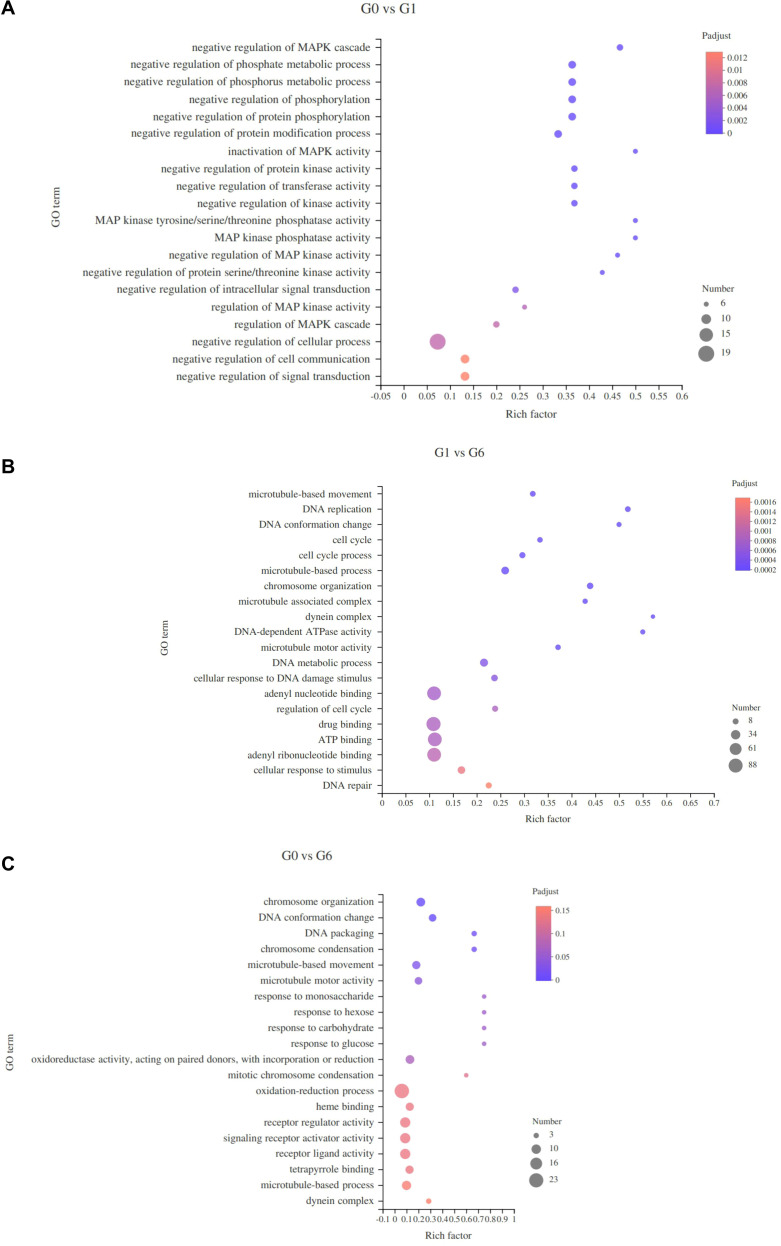
The most enriched GO terms of different groups. **(A)**, G0 vs G1; **(B)**, G1 vs G6; **(C)**, G0 vs G6. The y-axis represents enriched GO terms, and the x-axis represents the rich factor (the ratio of the number of genes enriched in a GO term to the total number of genes annotated to that term). Dot size indicates the number of differentially expressed genes, and dot color represents the adjusted *P* value.

The GO enrichment results of DEGs are summarized in **Table 5**. In the G0 vs G1 comparison, 282 GO terms were enriched, including 190 biological process terms, 72 molecular function terms, and 20 cellular component terms. The top 20 enriched GO terms are shown in **Figure 3A**. These terms were mainly associated with regulation of the MAPK cascade, negative regulation of phosphate metabolic processes, MAPK tyrosine/serine/threonine phosphatase activity, and MAPK phosphatase activity. These functions are closely related to MAPK signaling, which participates in the regulation of multiple cellular processes including cell proliferation, differentiation, apoptosis, and metabolic regulation. In the G1 vs G6 comparison, 348 GO terms were enriched, including 231 biological process terms, 93 molecular function terms, and 24 cellular component terms. The top enriched GO terms (**Figure 3B**) included microtubule-based movement, DNA replication, cell cycle, and DNA metabolic processes. For the G0 vs G6 comparison, 233 GO terms were enriched, including 123 biological process terms, 90 molecular function terms, and 20 cellular component terms. As shown in **Figure 3C**, enriched GO terms included DNA packaging and DNA conformational changes, as well as several terms associated with carbohydrate metabolism, such as response to monosaccharide, response to hexose, response to glucose, and response to carbohydrate.

**Table 5:** Summary of GO enrichment results for different comparisons.

Comparison	Total GO terms	Biological process	Molecular function	Cellular component
G0 vs G1	282	190	72	20
G1 vs G6	348	231	93	24
G0 vs G6	233	123	90	20

### 3.4 KEGG pathway enrichment analysis

**Figure 4:**
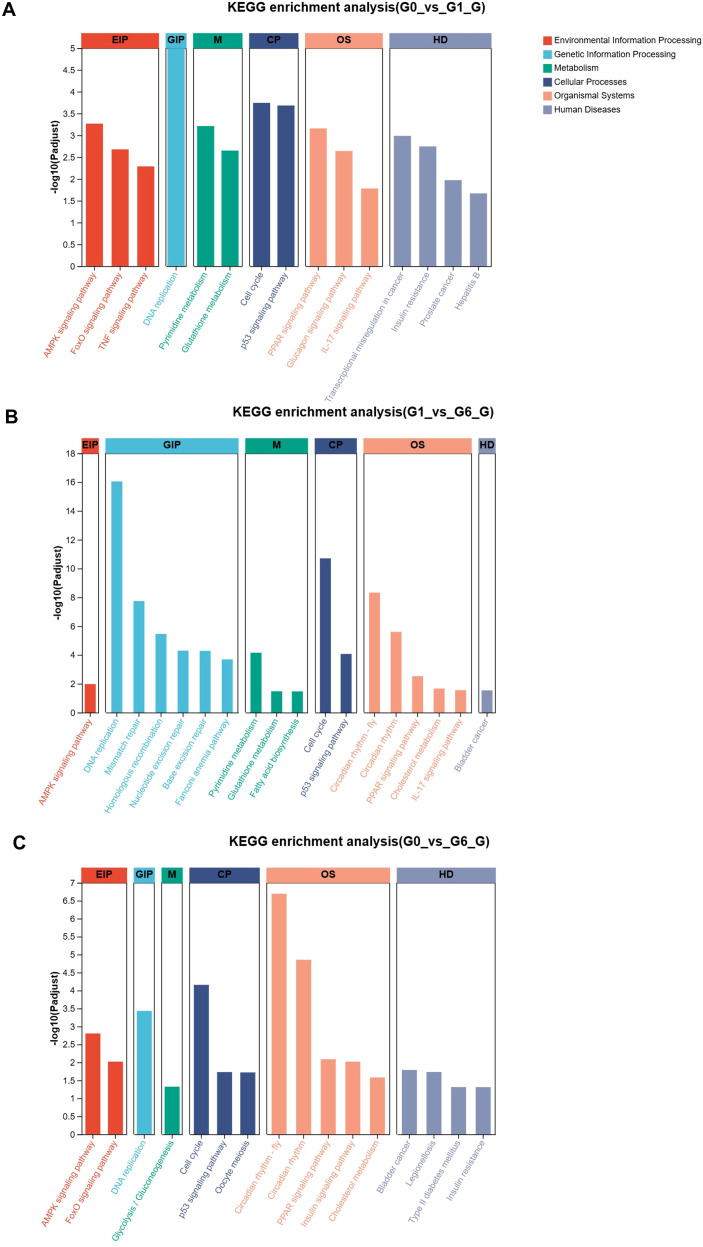
KEGG pathway enrichment analysis of differentially expressed genes (DEGs). **(A)**, G0 vs G1; **(B)**, G1 vs G6; **(C)**, G0 vs G6. The x-axis represents enriched KEGG pathways and the y-axis represents −log10(adjusted *P* value). Bar height indicates the enrichment significance of each pathway, and colors represent different KEGG functional categories.

Analysis revealed that DEGs from the G0 vs G1 comparison were enriched in 268 pathways, among which 23 pathways were significantly enriched. The top enriched pathways are shown in **Figure 4A**. These pathways included several metabolic signaling pathways associated with glucose regulation, such as the glucagon signaling pathway, insulin resistance, and FoxO signaling pathway. In addition, pathways related to energy and lipid metabolism, including the AMPK signaling pathway and PPAR signaling pathway, were also significantly enriched. Several pathways associated with cellular regulation were also identified, including the p53 signaling pathway and cell cycle. In the G1 vs G6 comparison, the top enriched pathways are presented in **Figure 4B**. Several pathways were associated with genetic information processing and cellular regulation, including DNA replication, mismatch repair, nucleotide excision repair, and cell cycle. In addition, several metabolic pathways related to lipid and energy metabolism were enriched, including fatty acid biosynthesis, cholesterol metabolism, the PPAR signaling pathway, and the AMPK signaling pathway. For the G0 vs G6 comparison, DEGs were significantly enriched in 16 KEGG pathways (**Figure 4C**), including several pathways associated with glucose metabolism, such as the FoxO signaling pathway, insulin signaling pathway, glycolysis/gluconeogenesis, type II diabetes mellitus, and insulin resistance pathways.

### 3.5 Expression patterns of glucagon-regulated metabolic genes

**Figure 5:**
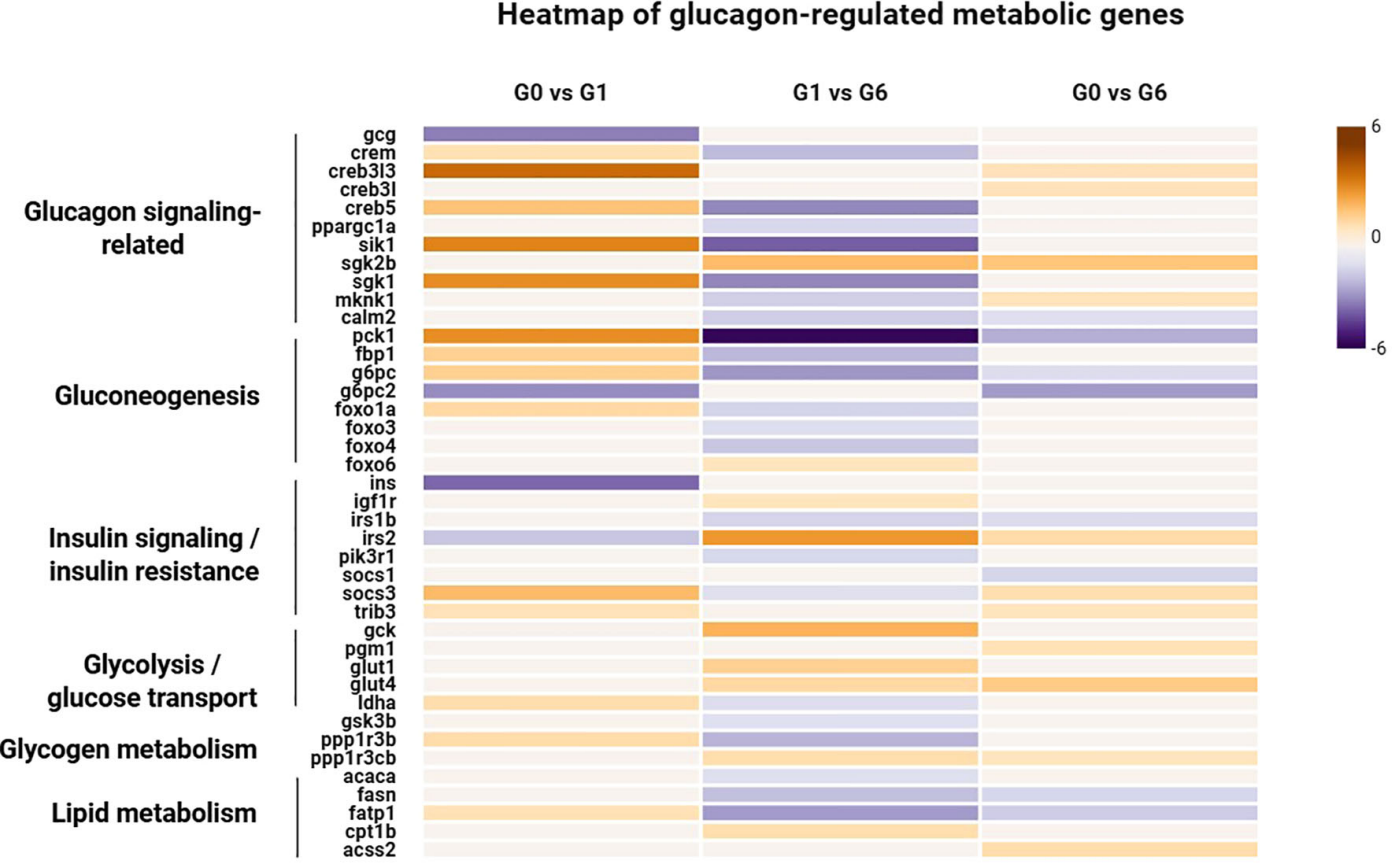
Heatmap of glucagon-regulated metabolic genes. log2FC of selected genes involved in glucagon signaling, gluconeogenesis, insulin signaling/insulin resistance, glycolysis/glucose transport, and glycogen and lipid metabolism are shown across three comparisons (G0 vs G1, G1 vs G6, and G0 vs G6). Orange indicates upregulated genes and purple indicates downregulated genes.

To further investigate the transcriptional changes of key metabolic genes in response to glucagon stimulation, a heatmap of selected genes involved in glucose and energy metabolism was constructed (**Figure 5**). These genes were mainly associated with glucagon signaling, gluconeogenesis, insulin signaling/insulin resistance, glycolysis/glucose transport, glycogen metabolism, and lipid metabolism. In the G0 vs G1 comparison, several genes associated with glucagon signaling and gluconeogenesis showed increased expression, including cAMP responsive element binding protein 3 like 3 (*creb3l3*), *creb5*, salt-inducible kinase 1 (*sik1*), serum/glucocorticoid regulated kinase 1 (*sgk1*), *pck1*, fructose-1,6-bisphosphatase 1 (*fbp1*), *g6pc*, and forkhead box protein O1a (*foxo1a*). In contrast, genes related to insulin signaling, such as insulin (*ins*) and insulin receptor substrate 2 (*irs2*), showed decreased expression levels. In addition, the regulatory genes *socs3* and *trib3* showed increased expression levels. In the G1 vs G6 comparison, the expression of several gluconeogenic genes decreased, while genes associated with glycolysis and glucose transport, including glucokinase (*gck*), and solute carrier family 2 members (*glut1* and *glut4*) were upregulated. Furthermore, the *socs3* displayed increased expression levels. In the G0 vs G6 comparison, genes associated with glucagon signaling, including *creb3l3* and *creb3l*, remained upregulated. In contrast, the insulin signaling-related gene insulin receptor substrate 1-B (*irs1b*) showed reduced expression. Meanwhile, genes involved in glycolysis and glucose transport like *gck* and *glut1* remained relatively upregulated, whereas several gluconeogenesis-related genes showed reduced expression compared with the early stage. In addition, the regulatory genes *socs3* and *trib3* displayed increased expression levels compared with the control group.

### 3.6 Validation of RNA-seq results by RT-qPCR

**Figure 6:**
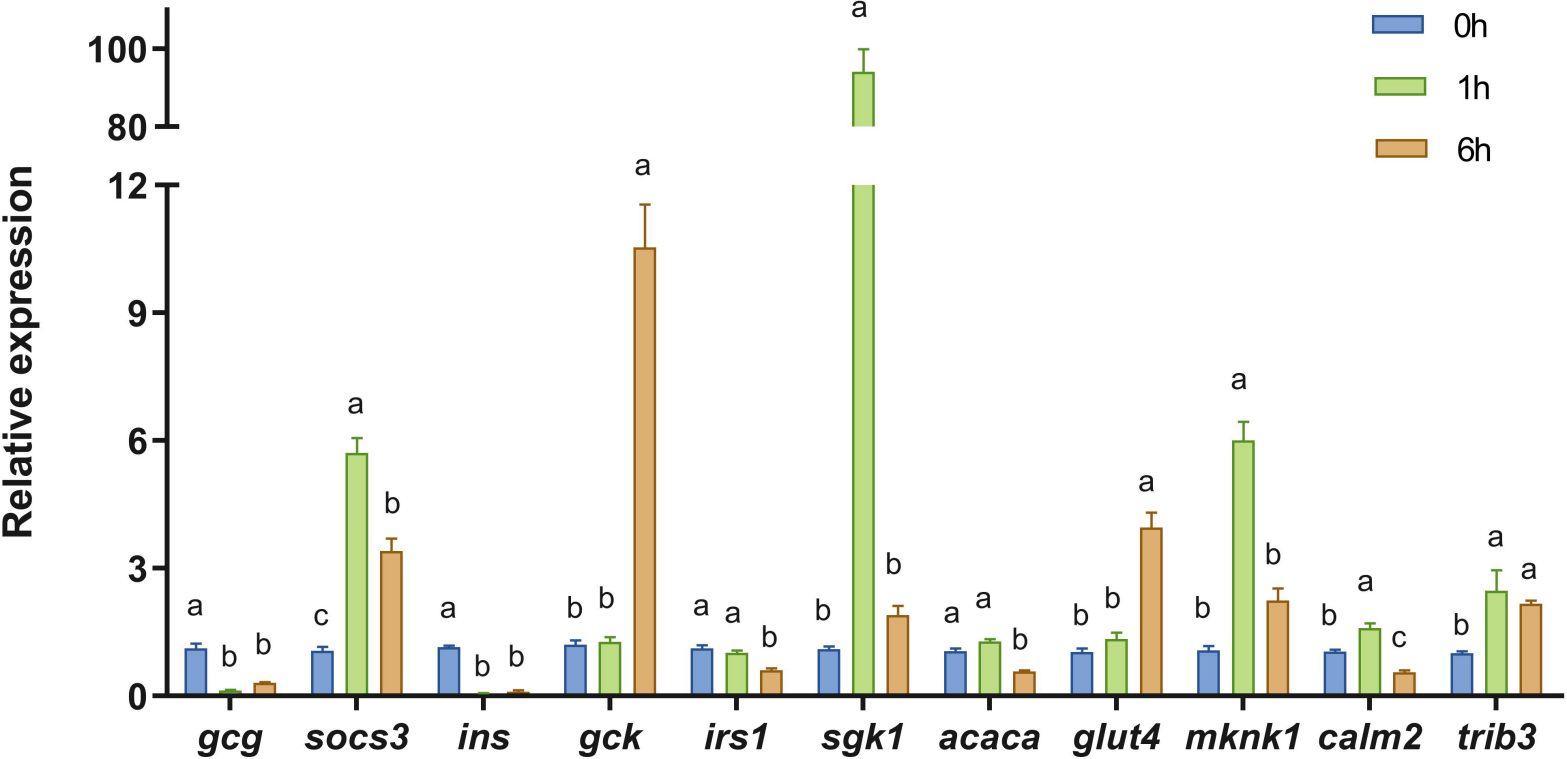
Validation of RNA-seq results by RT-qPCR. The relative expression levels of selected genes were measured in the liver of Japanese flounder at 0 h, 1 h, and 6 h following glucagon injection. Expression levels at 0 h were used as the control. Data are expressed as mean ± SE (n = 3). Different letters indicate significant differences among groups (*P* < 0.05). *gcg*, proglucagon; *socs3*, suppressor of cytokine signaling 3; *ins*, insulin; *gck*, glucokinase; *irs1*, insulin receptor substrate 1; *sgk1*, serum/glucocorticoid regulated kinase 1; *acaca*, acetyl-CoA carboxylase alpha; *glut4*, solute carrier family 2 member 4; *mknk1*, MAPK interacting serine/threonine kinase 1; *calm2*, calmodulin 2; *trib3*, tribbles pseudokinase 3.

11 DEGs were randomly selected for validation by RT-qPCR, including *gcg*, *socs3*, *ins*, *gck*, *irs1*, *sgk1*, acetyl-CoA carboxylase alpha (*acaca*), *glut4*, MAP kinase interacting serine/threonine kinase 1 (*mknk1*), calmodulin 2 (*calm2*), and *trib3*. As shown in **Figure 6**, the RT-qPCR results were consistent with the RNA-seq data.

### 3.7 Efficiency of TRIB3 gene knockdown

**Figure 7:**
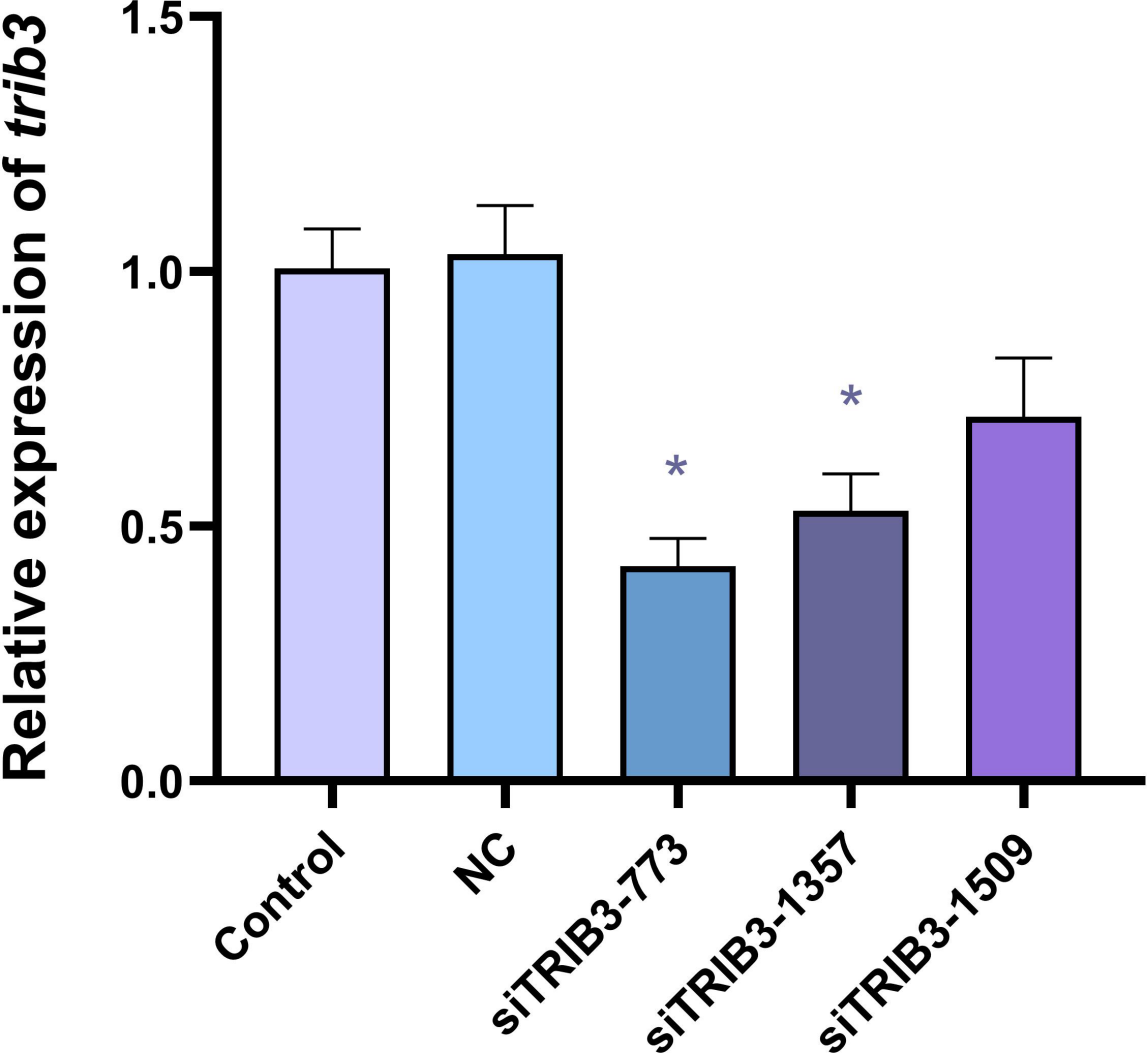
Knockdown efficiency of TRIB3 in hepatocytes transfected with different siTRIB3. Data are expressed as mean ± SE (n = 3). * represents significantly different compared with control group (*P* < 0.05).

As shown in **Figure 7**, compared with the control group, the negative control siRNA (NC) did not affect the expression of *trib3*. Among the three designed siRNAs, siRNA-773 showed the highest knockdown efficiency, reducing *trib3* mRNA expression by approximately 58%. Therefore, siRNA-773 was selected for subsequent experiments.

### 3.8 Effects of TRIB3 knockdown on gene expression and glucose production in hepatocytes under glucagon stimulation

**Figure 8:**
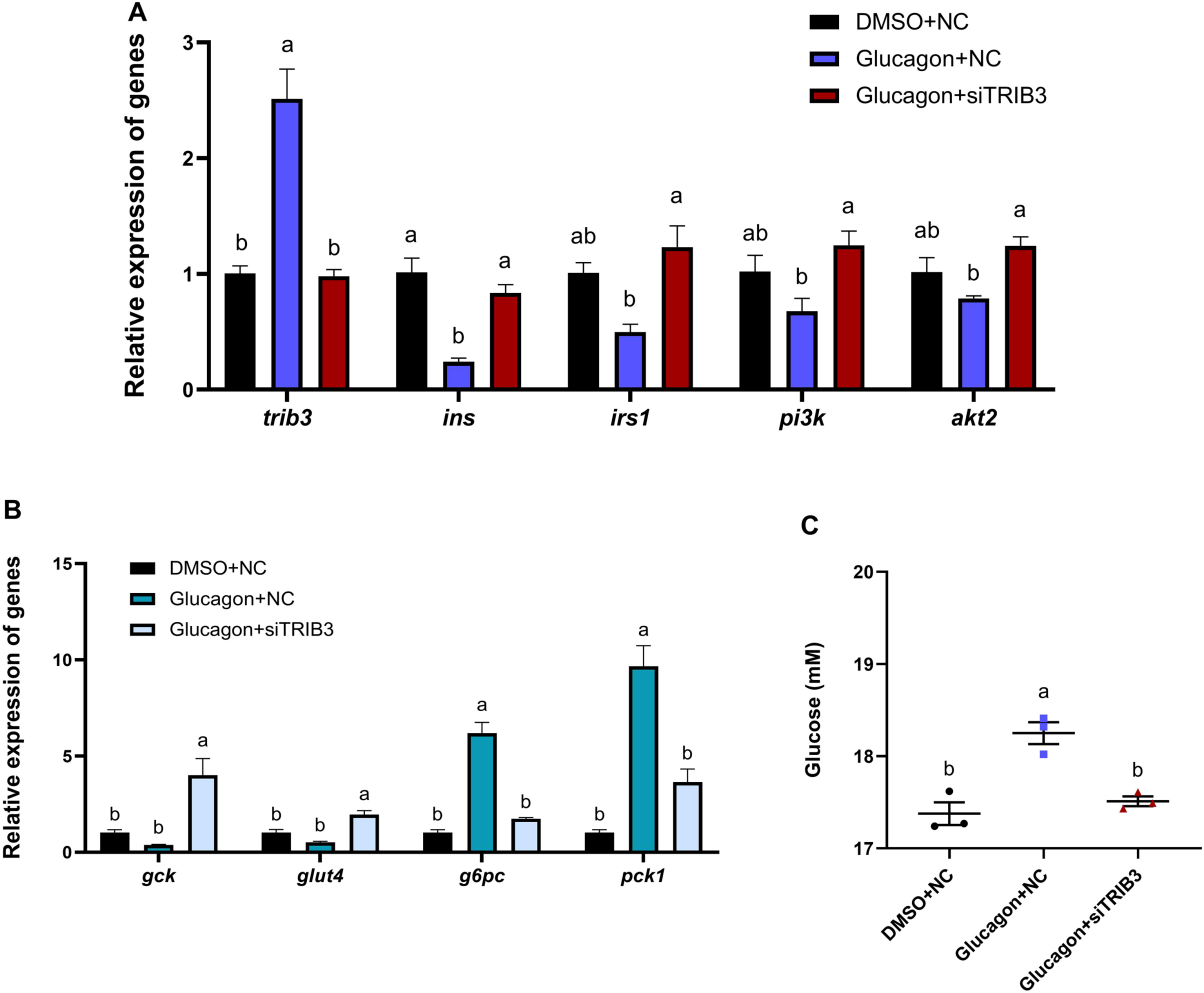
Effects of TRIB3 knockdown on gene expression and glucose levels in glucagon-treated Japanese flounder hepatocytes. **(A)**, Relative expression levels of *trib3*, *ins*, *irs1*, *pi3k*, and *akt2*. **(B)**, Relative expression levels of glucose metabolism-related genes (*gck*, *glut4*, *g6pc*, and *pck1*). **(C)**, Glucose concentration in the culture medium. Data are expressed as mean ± SE (n = 3). Different letters indicate significant differences among groups (*P* < 0.05).

As shown in **Figure 8A**, compared with the DMSO + NC group, glucagon treatment (Glucagon + NC) markedly increased the mRNA expression of *trib3* in Japanese flounder hepatocytes (*P* < 0.05). Meanwhile, the expression level of insulin signaling-related gene *ins* was significantly decreased (*P* < 0.05), while *irs1*, *pi3k*, and *akt2* showed a decreasing trend (*P* > 0.05). When *trib3* was knocked down during glucagon treatment, the mRNA expression of *trib3* was markedly reduced (*P* < 0.05), and the expression levels of *ins*, *irs1*, *pi3k*, and *akt2* were significantly upregulated compared with the glucagon-treated group (*P* < 0.05). As shown in **Figure 8B**, glucagon treatment significantly increased the expression of gluconeogenesis-related genes *g6pc* and *pck1* (*P* < 0.05), while the glycolysis- and glucose transport-related genes *gck* and *glut4* were downregulated (*P* > 0.05). Knockdown of *trib3* during glucagon treatment significantly reduced the expression of *g6pc* and *pck1* and increased the expression of *gck* and *glut4* (*P* < 0.05). In addition, as shown in **Figure 8C**, glucagon treatment significantly increased glucose concentration in the culture medium (*P* < 0.05), whereas *trib3* knockdown significantly reduced glucose levels compared with the glucagon-treated group (*P* < 0.05).

## 4 Discussion

This study systematically characterized the hepatic transcriptional responses of Japanese flounder to glucagon injection using RNA-seq analysis. In the G0 vs G1 comparison, several genes closely associated with glucagon signaling and gluconeogenesis-including *creb3l3*, *creb5*, *sik1*, *pck1*, *fbp1*, *g6pc*, and *foxo1a*-were significantly upregulated. These transcriptional changes indicate activation of the classical glucagon signaling cascade involving the cAMP/PKA pathway, which plays a central role in hepatic glucose production (Rodgers, 2022). Consistent with previous observations that plasma glucose levels in Japanese flounder increase significantly within 1 h after glucagon injection, these results further support the rapid activation of hepatic glucose production by glucagon (Yang et al., 2023). Interestingly, the transcription of the *gcg* gene was significantly reduced 1 h after glucagon injection. This pattern may reflect a feedback regulatory mechanism in which elevated circulating glucagon suppresses endogenous glucagon synthesis. Along with the activation of gluconeogenic genes, several genes associated with insulin signaling showed reduced expression. The mRNA levels of *ins* and *irs2* were significantly decreased at 1 h after glucagon injection, suggesting that glucagon may suppress hepatic insulin signaling during the early response phase. Similar interactions between glucagon and insulin signaling have been described in mammals, where glucagon attenuates insulin action by inhibiting the PI3K/AKT signaling pathway (Han et al., 2016; Rodgers, 2012; Liu et al., 2017). In rat models, acute glucagon infusion has been shown to induce insulin resistance in peripheral tissues (Patarrão et al., 2015). Although the opposing roles of glucagon and insulin in controlling hepatic glucose production are well established in mammals, relatively little information is available regarding their interaction in fish. Previous work by Yan et al. (2020) demonstrated that glucagon stimulates hepatic glucose production whereas insulin suppresses hepatic glucose output, indicating that these hormones exert counteracting effects on glucose metabolism. In the present study, glucagon injection was associated with reduced expression of insulin signaling-related genes, suggesting that glucagon may directly inhibit insulin signaling in the liver of Japanese flounder. Our previous studies have reported that glucagon administration results in increased circulating glucagon levels along with a decrease in plasma insulin levels in Japanese flounder (Yang et al., 2024), supporting the interaction between glucagon and insulin signaling pathways discussed in this study. Besides classical gluconeogenic genes, several metabolic genes associated with glucose and lipid metabolism were also differentially expressed following glucagon treatment. The upregulation of *ldha* may reflect enhanced lactate utilization, which can provide substrates for gluconeogenesis through the Cori cycle (Brooks, 2018). In addition, the elevated expression of fatty acid transport protein 1 (*fatp1*) suggests enhanced fatty acid uptake and lipid utilization, which may provide additional energy to support gluconeogenic processes (Anderson and Stahl, 2013; Rui, 2014). Consistent with this observation, KEGG enrichment analysis identified significant enrichment of pathways related to AMPK and PPAR signaling, both of which play central roles in regulating lipid metabolism and fatty acid oxidation (Hardie et al., 2012; Kersten, 2014). Taken together, these transcriptional responses suggest that glucagon injection triggers coordinated metabolic adjustments in the liver, involving gluconeogenesis, insulin signaling modulation, and lipid utilization.

In the G1 vs G6 comparison, the expression levels of several genes associated with glucagon signaling and gluconeogenesis-including *ldha*, *foxo1a*/*3*/*4*, *creb5*, PPARG coactivator 1 alpha (*ppargc1a*), *calm2*, *fbp1*, *g6pc*, and *pck1*-were significantly reduced, whereas the insulin signaling-related genes including *irs2* and *igf1r* showed increased expression. Furthermore, genes involved in glycolysis and glucose transport, including *gck*, *glut1*, and *glut4*, were upregulated. Together with previously reported changes in plasma metabolic parameters (Yang et al., 2024), these transcriptional patterns indicate that the metabolic effects of glucagon gradually decline several hours after injection. As glucagon signaling weakens, the transcription of gluconeogenic enzymes decreases, while glycolytic activity and glucose transport appear to increase. These results suggest a shift in hepatic metabolism from glucose production toward glucose utilization.

Despite the apparent attenuation of gluconeogenesis at 6 h, several transcriptional features indicate that the antagonistic interaction between glucagon and insulin signaling may still persist. In the G1 vs G6 comparison, *foxo6* expression remained elevated, whereas several important components of the insulin signaling pathway, *irs1b* and phosphoinositide-3-kinase regulatory subunit 1 (*pik3r1*), showed reduced expression. In addition, the expression of *ins* did not increase compared with the 1 h group, suggesting that insulin signaling had not fully recovered at this stage. Furthermore, when compared with the pre-injection condition (G0 vs G6), glucagon-responsive transcription factors such as *creb3l3* and *creb3l* remained upregulated, whereas *irs1b* expression remained suppressed. CREB family transcription factors mediate glucagon-induced transcriptional responses through the cAMP/PKA pathway, whereas IRS proteins function as key adaptor molecules in insulin signal transduction (Altarejos and Montminy, 2011; Myers and White, 1996). Therefore, although gluconeogenic gene expression declines at the later stage following glucagon injection, the persistent activation of glucagon-related transcription factors together with the suppression of IRS-mediated signaling suggests that glucagon may continue to exert inhibitory effects on hepatic insulin signaling. Such sustained antagonism between glucagon and insulin pathways may contribute to the maintenance of hepatic glucose output and could represent an important regulatory feature of glucose metabolism in carnivorous fish.

SOCS3 is widely recognized as a negative regulator of insulin signaling. Previous studies have shown that SOCS3 can inhibit IRS-mediated signal transduction and thereby attenuate downstream PI3K/AKT signaling (Jiang et al., 2012; Ueki et al., 2004; Xu et al., 2020). Previous studies in Japanese flounder have further shown that high-glucose-induced inflammatory responses can stimulate interleukin-6 secretion and subsequently upregulate *socs3* expression, which inhibited glucose transport through the PI3K/AKT pathway (Deng et al., 2018). In addition, knockdown of SOCS3 has been reported to enhance the sensitivity of the insulin pathway, thereby promoting glucose transport in Japanese flounder (Qin et al., 2026). In mouse hepatocytes, glucagon treatment has been reported to increase *socs3* mRNA levels by approximately fourfold (Gaudy et al., 2010). In the present study, *socs3* expression increased significantly 1 h after glucagon injection and declined at 6 h relative to the 1 h group, although it remained higher than the pre-injection level. This expression pattern suggests that SOCS3 may contribute to glucagon-induced suppression of insulin signaling and may remain involved in this regulatory process during the later stage of the response. More importantly, transcriptome analysis identified TRIB3 as another gene that was markedly upregulated following glucagon injection. TRIB3 is a stress-responsive gene involved in multiple signaling pathways and has been associated with pancreatic β-cell apoptosis and insulin resistance, making it an important molecular factor in type 2 diabetes (Okamoto et al., 2007). In human hepatocellular carcinoma cells, TRIB3 promotes hepatic glucose production by inhibiting AKT phosphorylation (Du et al., 2003). In the present study, the upregulation of *trib3* after glucagon injection suggests that TRIB3 may participate in glucagon-mediated metabolic regulation by attenuating insulin signaling in liver. Functional experiments further supported this hypothesis. Under glucagon incubation conditions, knockdown of TRIB3 significantly reduced glucose levels in the hepatocyte culture medium and increased the expression of genes associated with insulin signaling. In addition, genes involved in glucose utilization tended to increase, whereas gluconeogenesis-related genes showed a decreasing tendency after TRIB3 knockdown. These results indicate that TRIB3 may contribute to glucagon-induced metabolic regulation by influencing the balance between hepatic glucose production and utilization. Although the phosphorylation status of AKT was not tested in the present study, the combined transcriptomic results and functional interference experiments suggest that TRIB3 may play a vital role in glucagon-induced inhibition of the PI3K/AKT signaling pathway.

## 5 Conclusion

In conclusion, glucagon regulates hepatic glucose metabolism in Japanese flounder through multiple signaling pathways. Transcriptome analysis indicated that glucagon promotes gluconeogenesis while potentially suppressing insulin signaling through regulatory molecules such as SOCS3 and TRIB3. Functional experiments further showed that knockdown of TRIB3 activated PI3K/AKT pathway under glucagon treatment, supporting its role in glucagon-mediated metabolic regulation. These findings provide novel insights into the endocrine mechanisms underlying glucose metabolism and may help explain the relatively low carbohydrate utilization capacity observed in carnivorous fish.

## Data Availability

The raw sequencing data in this study have been deposited in the NCBI Sequence Read Archive (SRA) under the BioProject accession number PRJNA1425731.
